# Relationship between carotid intima‐media thickness (cIMT) and dual‐system imbalance in tobacco dependence: An rs‐fMRI research

**DOI:** 10.1002/brb3.3059

**Published:** 2023-06-12

**Authors:** Qiaowen Tan, Shaoke Li, Mengqian Yu, Jingyi Zhao, Xiaosa Chi, Yan Han, Zongjun Guo

**Affiliations:** ^1^ Department of Gastroenterology Affiliated Hospital of Qingdao Binhai University Qingdao Shandong Province China; ^2^ Department of Geriatrics Affiliated Hospital of Qingdao University Qingdao Shandong Province China; ^3^ Department of Medical Imaging Affiliated Hospital of Qingdao University Qingdao Shandong Province China; ^4^ Medical Research Center The Affiliated Hospital of Qingdao University Qingdao Shandong Province China

**Keywords:** carotid intima‐media thickness, goal‐directed network, habitual network, resting‐state functional magnetic resonance imaging, tobacco dependence

## Abstract

**Background and purpose:**

According to the classic cognitive behavioral theory proposes, dysfunctional goal‐directed and habit control systems are considered central to the pathogenesis of dependent behavior and impair recovery from addictions. The functional connectivity (FC) of the brain circuits for goal‐directed or habitual behavior has not been clearly reported in tobacco‐dependent groups. Smoking is one of the factors in the formation of atherosclerosis. Studies have shown that the thickness of carotid intima‐media (cIMT) is associated with attention‐executive‐psychomotor functioning. Therefore, we hypothesized whether cIMT in tobacco‐dependent individuals is associated with changes in the FC of the dual‐system network.

**Methods:**

A total of 29 male tobacco‐dependent subjects (tobacco‐dependent group) (mean age: 64.20 years, standard deviation [SD]: 4.81 years) underwent resting‐state functional magnetic resonance imaging (rs‐fMRI). Exactly 28 male nonsmokers (control group) (mean age: 61.95 years, SD: 5.52 years) were also recruited to undergo rs‐fMRI. We used the dorsolateral striatum (putamen) and dorsomedial striatum (caudate) as regions of interest for whole‐brain resting‐state connectivity to construct habitual and goal‐directed brain networks, respectively. In addition, all participants were evaluated by carotid artery ultrasound to obtain the cIMT values. Then, we compared the dual‐system brain networks between the tobacco dependence and control groups and the relationship between cIMT and imbalance of dual‐system brain networks in tobacco dependence.

**Results:**

The results showed a reduction in the connection between the caudate and precuneus and an increased connection between the putamen and prefrontal cortex; and supplementary motor area. The bilateral connectivity between the caudate and inferior frontal gyrus showed a significant negative correlation with the cIMT, and no positive correlation was observed with cIMT in the brain region that connects to the caudate. However, for the putamen, increased connectivity with the inferior temporal and medial frontal gyri was strongly associated with a high cIMT.

**Conclusions:**

The results indicate that the formation of tobacco dependence behavior is related to changes in the dual‐system brain network. Carotid sclerosis is associated with the weakening of the goal‐directed network and enhancement of the habit network in tobacco dependence. This finding suggests that tobacco dependence behavior and clinical vascular diseases are related to changes in brain functional networks.

## Graphical abstract

we evaluated the conversion of dual‐system networks functional connectivity after tobacco dependence and the relationship between the thickness of the carotid Intima‐Media (cIMT) and the conversion of dual‐system networks functional connectivity

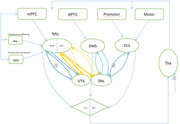

1

Smoking is one of the major public health problems worldwide and the main cause of preventable death in most countries. Although most individuals with tobacco dependence hope to stop, only a handful succeed. Given that tobacco dependence is a chronic brain disorder characterized by forced tobacco use, smokers may lose control of their smoking behavior (Berrendero et al., 2010). A theory has been postulated to explain the formation mechanism of dependent behavior that dysfunctional goal‐directed and habit control systems are central to the pathogenesis of dependent behavior and impaired recovery from addictions (Hyman et al., 2006; Kalivas & O'Brien, 2008). Goal‐directed behavior refers to the actions that are performed to achieve the desired goal. When action is taken on certain regularity, habits are formed. Habits can facilitate actions that do not require planning and can lead to great efficiency, but they are often inflexible. Cognitive behavioral theory explains that dependent behavior is a learning disorder; the transition from casual to habitual behavior is associated with reduced goal‐directed behavior, and strengthened habitual behavior (Everitt & Robbins, 2013; Linnebank et al., 2018).

Nicotine, the major reinforcing component of tobacco smoke, acts in the brain through neuronal nicotinic acetylcholine receptors (nAChRs). In addition to playing a key role in the behavioral actions of nicotine, which contribute to the development of tobacco dependence, nAChRs also affect the brain circuitries involved in reinforcement, mood, attention, and food consumption (Picciotto & Kenny, 2021). Relevant research demonstrated that smokers differ from nonsmokers in terms of their regional brain structure and neurochemistry and activation in response to smoking‐related stimuli and during the execution of cognitive tasks (Azizian et al., 2009). Bruijnzeel's study indicated that acute nicotine administration activates brain areas involved in reward signaling, compulsive drug intake, and motor function. His study also showed that acute nicotine administration leads to the activation of the striato‐thalamo‐orbitofrontal circuit, which plays a role in compulsive behavior. Nicotine‐induced dysregulation of this brain circuit may contribute to the development of compulsive smoking (Bruijnzeel et al., 2014). Contemporary neuroscience aims to understand how neuronal activity produces internal processes and observable behavioral states. This aim crucially depends on network‐based analyses of the working brain given that behavioral states arise from information flow and connectivity within and between discrete and overlapping brain regions, which form brain networks. Functional magnetic resonance imaging (fMRI) offers a key to advancing brain network neuroscience. fMRI measures inter and intraregional circuits at behaviorally relevant spatial temporal resolution. Here, we propose that brain networks obtained from clinical fMRI models can provide a better understanding of cross‐sectional observations of populations.

The functional connectivity (FC) of the brain circuits for goal‐directed or habitual behavior has not been studied previously in tobacco dependence groups. Using fMRI, several studies investigated the brain response to smoking‐related stimuli. Baker et al. (2017) observed that compared with monetary rewards, cigarette rewards showed a higher reward response in the tobacco dependence group. This finding suggests that cigarette addicts show deficits in the recruitment of brain reward pathways (Lin et al., 2020). A study of the neurological effects of “smoking” electronic cigarettes using task‐state fMRI showed brain activation in the motor cortex, cingulate cortex, putamen, thalamus, globus pallidum, and cerebellum and relative inactivation of the ventral striatum and orbitofrontal cortex (Wall et al., 2017). All these studies showed that overreliance on habitual system contributes to the development of tobacco dependence behavior.

Cigarette smoking is a major risk factor for atherosclerosis and other cardiovascular diseases (Ockene & Miller, 1997). Substantial evidence supports the promoting effect of nicotine on atherosclerosis in a long‐term basis although short‐term exposure to nicotine is considered relatively harmless (Heeschen et al., 2001). Increasing pieces of evidence have demonstrated that nicotine impairs the cardiovascular system by targeting vascular endothelial cells, but the underlying mechanisms remain obscure. Brain networks change in normal aging and cognitive disorders, Parkinson's, and autoimmune diseases (Chung et al., 2020; Filippi et al., 2020; Srivishagan et al., 2020; Valdés Hernández et al., 2021). Intimal‐medial thickening (IMT) of the carotid wall is an accepted peripheral marker of atherosclerosis. It is associated with the increased risk for myocardial infarction and stroke and low attention‐executive‐psychomotor functioning. Haley's research illustrated that the blood oxygenation level‐dependent contrast is highly sensitive to peripheral vascular health as measured by IMT. He demonstrated that a high IMT was associated with signal intensity in the right middle frontal gyrus (Haley et al., 2007). Sweet's research showed that normal performance of a challenging verbal working memory task among high‐functioning multiple sclerosis patients was associated with a shift toward greater activity in regions related to sensorimotor functions and anterior attentional/executive components of the verbal working memory system (Sweet et al., 2004). Therefore, we hypothesized whether the strength of target and habitual brain network connectivity is correlated with carotid intima thickness in smokers.

The putamen and caudate are core regions of habitual and goal‐directed networks (Tricomi et al., 2009; van der Straten et al., 2020; Watson et al., 2018). The dysfunction of both structures is considered the neuroanatomical basis of dependence (Tau et al., 2014). However, the dual‐system theory does not explain the typical clinical observations of carotid atherosclerosis associated with cognition and decision‐making (van der Flier et al., 2018). Sojkova et al. (2010) discovered that a high carotid intima‐media thickness (cIMT) was associated with a low regional cerebral blood flow in lingual, inferior occipital, and superior temporal regions in older adults. Brutto pointed out that the association between increased cIMT and cognitive dysfunction is mostly mediated by the increase in age (Del Brutto et al., 2020). Furthermore, studies on healthy aging humans have shown that aging affects controlled and conscious processing that impairs goal‐directed ability (de Wit et al., 2014; Span et al., 2004; Tomás et al., 2016; Worthy et al., 2011). Elijah used novel tablet‐based pointing tasks to prove the greater effect of age on response time in goal‐directed tasks compared with that in habitual tasks (Li et al., 2018). However, aging and atherosclerosis studies showed that atherosclerosis is a marker of cellular senescence (Wang & Bennett, 2012). Based on the above research, we hypothesized the possible transformation in the two‐system brain network in tobacco‐dependent people and association of cIMT with the transformation of the two‐system brain network. However, this new model that connects the cIMT with the neuroscience‐inspired dual‐system theory of dependent behavior remains to be tested.

In this study, we investigated the relationship of cIMT with resting‐state FC in the caudate, which serves as the primary center of the goal‐directed network, and in the putamen, which serves as the primary center of the habit network. Meanwhile, we compared the dual‐system brain networks between the tobacco dependence and control groups. We hypothesized that comparative studies of tobacco dependence would reveal the neuroimaging mechanisms about the disequilibrium between goal‐directed and habit control networks (prefrontal cortex (PFC)/caudate and premotor cortex/putamen, respectively) (de Wit et al., 2012; Delorme et al., 2016; Yamagata et al., 2012). Specifically, we aimed to determine whether connectivity is reduced within the goal‐directed network and increased within the habit network. According to previous studies, cIMT affects the cognitive decision‐making ability of the elderly. Thus, we hypothesized that this transition is related to atherosclerosis.

## 2 MATERIALS AND METHODS

### 2.1 Participants

A total of 62 participants were enrolled in the study through the Affiliated Hospital of Qingdao University. Two participants withdrew from the experiment, and three fMRI datasets were not usable due to more than 1.5° of head movement. The remaining 29 participants in the smoking group were 55–75‐year old (mean age: 64.20 years, standard deviation [SD]: 4.81 years), and 28 participants in the control group had a range of 55–75 years (mean age: 61.95 years, SD: 5.52 years). They all participated in this study (Table 1 shows other participant characteristics). The ethics committee of Qingdao University Medical College approved this study, and all participants signed an informed consent. All the participants met the following inclusion criteria: ⑴ no contraindications to MRI; ⑵ no brain disease (space‐occupying lesion, infarction, or ischemic focus) or other medical conditions (heart, lung, liver, and kidney); ⑶ no cognitive impairment and IQ scores of more than 90 points; ⑷ meeting the diagnostic criteria of the fourth edition of the Diagnostic and Statistical Manual of Mental Disorders, that is, daily smoking volume of not less than 10 cigarettes and smoking age of not less than 2 years or less than 3 months in the last year (Vieta, 2016); ⑸ no other history of addiction; ⑹ can complete the questionnaire; ⑺ have completed the Fagerstrom Test for Nicotine Dependence (FTND) (Heatherton et al., 1991).

**TABLE 1:** Demographic data, psychological data (*x* ± *s*)

	Tobacco dependence group (*n* = 29)	Control group (*n* = 28)	*p*
Age	64.20 ± 4.81	61.95 ± 5.52	.18
Length of education (year)	14.40 ± 3.59	14.40 ± 3.59	.63
IQ	95.55 ± 2.39	94.60 ± 3.00	.28
MMSE	29.35 ± 0.81	29.35 ± 0.81	.69
HAMA	3.10 ± 1.55	3.15 ± 1.79	.92
HAMD	2.95 ± 1.93	2.90 ± 1.68	.93
cIMT (mm)	0.96 ± 0.36	0.80 ± 0.48	.03

*Note*: Means (standard deviations) are presented.

### 2.2 Procedure

All participants underwent an interview for psychiatric screening by completing the FTND scale, and psychometric assessment was carried out by a trained geriatrician prior to the fMRI scan. The mental cognitive assessment included (1) MMSE for rapid screening of cognitive function, (2) IQ test, and (3) Hamilton Depression Scale (HAMD), and Hamilton Anxiety Scale (HAMA). Then, the participants lay on the examination bed awake and at rest, with their eyes closed and them breathing calmly for the resting‐state fMRI (rs‐fMRI). Afterward, they were examined by carotid ultrasonography.

### 2.3 MRI scanning parameters and conditions

All participants lay on the examination bed awake and at rest, with their eyes closed and calm breathing. Scanning was performed with the GE3.0T HDX superconducting whole‐body MRI system. The parameters for three‐dimensional magnetization preparation fast gradient‐echo sequence scan for T1‐weighted images were as follows: parameter TE = 2.3 ms; prep time = 450 ms; FA = 15°; bandwidth = 19.23; FOV = 23 mm × 23 mm; layer thickness = 1.0 mm; pitch = 0 mm; matrix = 230 × 230; NEXl.00, 178 floors. The parameters for single excitation of fMRI and gradient‐echo planar imaging scan for T2‐weighted images were as follows: parameter TE/TR = 30/2000 ms; layer thickness = 4.0 mm; layer spacing = 0 mm; matrix = 64 × 64; FA = 900; FOV = 23 mm × 23 mm; NEX = 1; 45 scan layers; scan time: 5 min and 10 s.

### 2.4 Carotid ultrasonography

All the subjects were examined by color Doppler ultrasonography (GE LOGIQ E9) by ultrasound physicians in our hospital. Evaluation was performed in the supine position in a dark and quiet room. After both sides of the common carotid artery were maximized in the longitudinal plane, the cIMT was measured at the far wall of the common carotid artery about 1–1.5 cm from the bifurcation. cIMT measurement was obtained by averaging the distance between the lumen‐intima and media–adventica interface. The mean value of three bilateral measurements was considered the final cIMT value.

### 2.5 Data analysis

#### 2.5.1 fMRI data

Matlab 2010b platform (Matrix Laboratory, http://www.verycd.com/topics/2848827), which includes SPM 8 (http://www.fil.ion.ucl.ac.uk/spm), DPARSF 2.1 (http://rfmri.org/DPARSF), Rest 1.6 (http://www.restfmri.net), and BrainNet Viewer software package (NITRC: BrainNet Viewer: Tool/Resource Info), was used for all rs‐fMRI data processing. We calculated the fractional amplitude of low‐frequency fluctuations and functional connection value and accomplished a brain map presentation. Image preprocessing included space normalization, time correction, smoothing, head movement correction, and filtering. All functional images were then smoothed using a 4 mm Gaussian kernel. The sampling rate was 3 × 3 × 3 mm^3^, the time series in each session was band‐pass filtered (0.01–0.08 Hz), and linear drift removal was performed. All images automatically formed a data file after preprocessing. If the head of a participant moved by more than 1.5 mm or rotated by more than 1.5° on any of *x*‐, *y*‐, or *z*‐axis, the participant was excluded.

##### 2.5.1.1 Brain map

Whole‐brain resting‐state connectivity was examined using 6 mm‐radius spherical seeds centered on dorsolateral striatum (putamen DLS) and dorsomedial striatum (caudate DMS) regions of interest (ROIs). We used the Rest 1.6 software to conduct one‐sample *t* test for the smoking and control groups and obtained the FC activation map of the whole brain by the BrainNet Viewer software package (*p* < .01; the whole‐brain default mask was used, with cluster≥19 voxels and corrected by AlphaSim (*p* < .05)). Figure 3 presents the three‐dimensional brain maps. The green dots and lines represent the control group, and the red ones represent the smoking group. The yellow dots indicate the ROIs. The size was set based on the *t* value of each brain area, and the name was also marked.

##### 2.5.1.2 FC data

Based on the evidence showing that the caudate and putamen participate in behavior execution, we tested our previous hypothesis that the impairment of cognitive domains is related to the imbalance of whole‐brain functional connection of the putamen and caudate in tobacco dependence. We tested the differences in FC of a priori anatomical ROIs between smoking and control subjects based on the known neurobiological profile of habit and goal‐directed and previous findings in the literature (van der Straten et al., 2020; Watson et al., 2018). Whole‐brain resting‐state connectivity was examined using 6 mm‐radius spherical seeds centered on putamen DLS and caudate DMS ROIs. The coordinates were automatically provided by the AFNI‐supplied atlas, caudate (15, 12, 9), and putamen (28, 5, 2) (Harrison et al., 2009). fMRI denoised data were inputted to DPARSF 2.1 to estimate the FC. The mask of the two‐sample *t* test was formed by the two sets of brain areas obtained by single‐sample *t* test, and the formula was (*i*1 + *i*2) (*p* < .01; threshold = 2.7116, degrees of freedom (df) = 55, cluster ≥ 19 voxels, corrected by AlphaSim (*p* < .05)). Fisher's *r*‐to‐*z* transform was conducted for the appropriate df.

##### 2.5.1.3 Carotid ultrasound data

The unbalanced functional connection between the putamen and caudate nucleus causes the impaired cognitive domain in tobacco dependence, and this damage can lead to diseases (e.g., arteriosclerosis). To study the neuroimaging mechanism of arteriosclerosis, we performed a correlation analysis using the ROI approach to assess whether the strength of functional connection in preselected ROIs (based on fMRI results) is associated with cIMT (significant threshold is corrected with *p* < .05, and cIMT is different among groups).

Statistical processing of data was carried out in SPSS software. We applied Shapiro–Wilk's test to the normality test. The data with normal distribution were expressed as $\bar{x}\pm s$ and analyzed by *t* test. The skewed distribution data were represented by the median and analyzed by Mann–Whitney rank‐sum test.

## 3 RESULT

### 3.1 Clinical data

A total of 29 tobacco‐dependent subjects and 28 nonsmokers participated in our study (Table 1). No significant differences were observed in age, educational level, IQ, MMSE, HAMA, and HAMD results across the two groups (*p* > .05). The cIMT value was significantly higher in the smoking group than the control group (*p* = .03).

### 3.2 FC results

**FIGURE 1:**
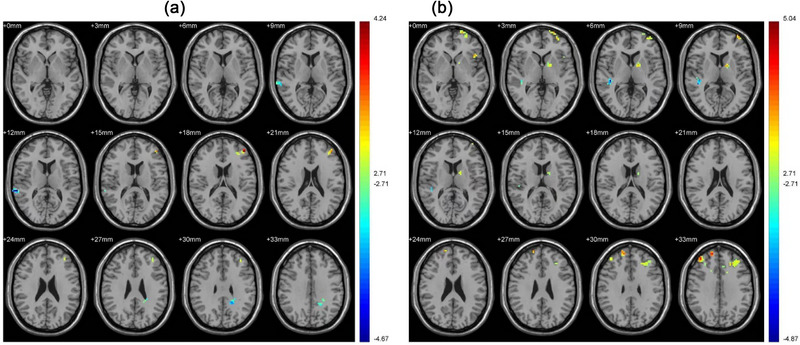
(A) The images of changes in goal‐directed brain network (the regions of interest [ROI] in caudate) compared between the smoking group and the control group; (B) the images of changes inhabit brain network (the ROI in putamen) compared between the smoking group and the control group. Blue to red indicates that the activation level gradually increased. *p* < .01, cluster ≥ 19 voxels, and corrected by AlphaSim.

For the ROI in caudate, compared with the control participants, the smoking participants exhibited significant a positive correlation with left medial frontal gyrus (MFG) (BA10) and a negative correlation with the right superior temporal gyrus (STG) (BA22) and left precuneus (PCUN) (BA31) (Table 2 and Figure 1A).

**TABLE 2:** Comparison of functional connectivity (FC) between the smoking and control groups

Seed	Connected region	Brodmann partition	Clusters	Peak MNI (peak coordinates)	*t*
Caudate	Tobacco dependence group > Control group				
	Left middle frontal gyrus	10	27	−45 45 18	4.07
Tobacco dependence group < Control group					
	Right superior temporal gyrus	22	19	63 −36 12	−4.67
	Left parietal lobe	31	20	−18 −45 30	−3.71
Putamen Tobacco dependence group > Control group					
	Left inferior frontal gyrus	47	21	−45 21 −3	3.90
	Left superior frontal gyrus	10	38	−24 69 3	3.76
	Left lateral globus pallidus	/	29	−15 0 9	3.71
	Right superior frontal gyrus	9/10	20	15 54 33	4.41
	Left cingulate gyrus	9	156	−18 30 39	4.20
	Right middle frontal gyrus	9	104	27 27 39	5.04
	Left middle frontal gyrus	8	29	−48 9 48	4.71
	Left superior frontal gyrus	8	56	−9 39 51	4.66
Tobacco dependence group < Control group					
	Right temporal lobe	41	26	42 −30 6	−4.87
	Right postcentral	40/5	36	21 −42 60	−4.49

*Note*: *p* < .01, cluster ≥ 19 voxels, corrected by AlphaSim.

The ROI in putamen exhibited positive correlations with the PFC (BAs 9, 10, and 47), supplementary motor area (SMA) (BA 6, 8), left lateral globus pallidus, and left cingulate gyrus (BA 31) and negative correlations with the right temporal lobe (BA 41) and right postcentral (BA 5) (Table 2 and Figure 1B).

### 3.3 Carotid ultrasound results

The cIMT mean value of the smoking group was 0.96  ±  0.36 mm, that of the control group was 0.80 ± 0.48 mm. Smoking participants had higher cIMT values than the control group (*p* = .03, Table 1).

### 3.4 Associations between cIMT and FC strength

**FIGURE 2:**
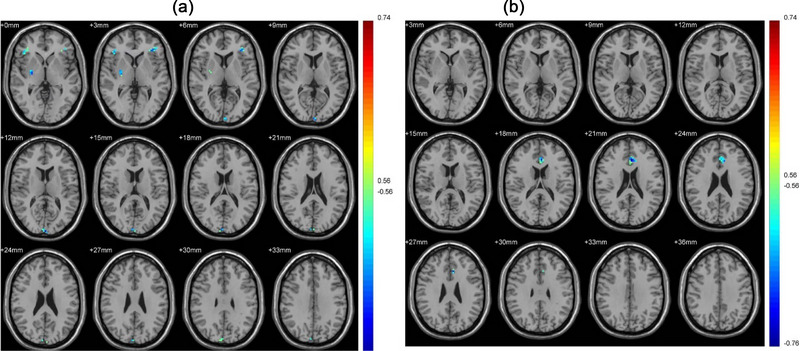
(A) The images of the association between carotid intima‐media (cIMT) values and goal‐directed brain network in the smoking group; (B) the images of the association between carotid intima‐media (cIMT) values and habit brain network in the smoking group. Blue to red indicates that the activation level gradually increased. *p* < .01, cluster ≥ 19 voxels, and corrected by AlphaSim.

In the tobacco dependence group, a higher cIMT was associated with the reduced FC between the caudate and several brain regions, including the right putamen and inferior frontal gyrus, bilaterally (Table 3 and Figure 2 A). However, an increased connectivity was observed between the putamen, inferior temporal gyrus, and MFG (Table 3 and Figure 2B). The results showed that a high cIMT was associated with decreased FC of the goal‐directed brain network and increased FC of the habit brain network.

**TABLE 3:** Correlations between the carotid intima‐media (cIMT) and the two brain network (goal and habit) functional connectivity (FC) in the smoking group

Seed	Region	Brodmann partition	Clusters	Peak MNI (peak coordinates)	*r*
Caudate	Positively correlated				
	None	/	/	/	/
	negative correlation				
	Right putamen	/	20	27 −9 −3	−.76
	Right inferior frontal gyrus	/	19	42 30 3	−.65
	Left Inferior Frontal Gyrus	/	21	−36 36 6	−.68
	Right cuneus	19	28	0 −99 12	−.70
Putamen	Positively correlated				
	Left Inferior Temporal Gyrus	20	23	−45 0 −45	.69
	Right medial frontal gyrus	25	21	3 30 −15	.74
	negative correlation				
	Left anterior cingulate	24	42	−3 30 21	−.76

*Note*: *p* < .01, cluster ≥ 19 voxels, corrected by AlphaSim.

**FIGURE 3:**
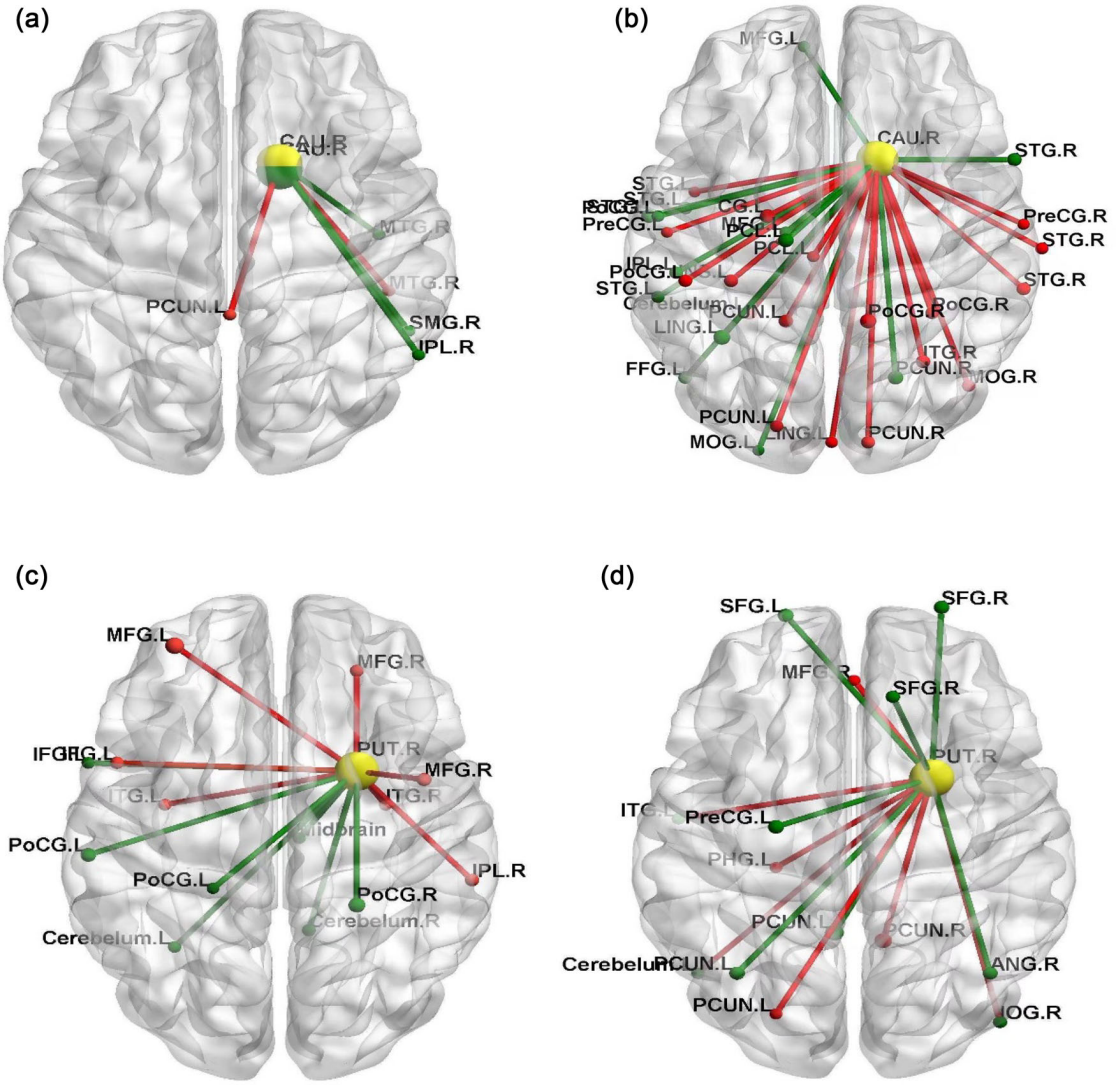
Brain functional connection map: (A) shows increased functional connections between the whole brain and caudate nucleus in both groups; (B) shows the reduction functional connections between the whole brain and caudate in both groups; (C) shows increased functional connections between the whole brain and putamen in both groups; and (D) shows the reduction functional connections between the whole brain and putamen in both groups. The green dots and lines represent the control group, and red represents the smoking group. The yellow represents the regions of interest [ROI]. The size of the node was set according to its *t* value. The larger the value, the greater the activation intensity. *p* < .01, cluster ≥ 19 voxels, and corrected by AlphaSim.

## 4 DISCUSSION

In the present study, we examined the relationship between tobacco‐dependent behavior, cIMT, and FC in tobacco‐dependent individuals to test whether the formation of smoking dependence behavior is related to a shift from the goal‐directed to the habit network and whether the accelerated aging of arteries is associated with this transition. Using a seed‐to‐voxel resting‐state analysis, we observed that in the tobacco dependence group, the strengths of functional connections were reduced between seeds (caudate) and left PCUN (BA31) and right STG (BA22) in the goal‐directed network and increased between seeds (caudate) and the left MFG (BA10). The activity of the goal‐directed system was negatively correlated with the cIMT value. In habit brain network of the tobacco dependence group, the strength of functional connections increased between seeds (putamen) and several brain regions, such as PFC (BAs 9, 10, and 47), SMA (BA 6,8), left lateral globus pallidus, and left cingulate gyrus (BA 32), and reduced between seeds and the right temporal lobe (BA 41) and right postcentral (BA 5). The activity of the habit system was positively correlated with the cIMT value. All in all, these results suggest that tobacco dependence behavior is primarily associated with reduced functional activity of the goal‐directed network and increased functional activity in the habit network. Accelerated aging of arteries is associated with this transition.

Dual‐system theory assumes that addiction behavior, such as smoking, may be caused by the imbalance between goal‐directed and habitual networks (Woodhead & Robbins, 2017). According to previous studies, drug dependence behavior may be related to a reduced ability for dorsolateral PFC (dlPFC) to exert goal‐directed control (Smith & Laiks, 2018). However, no evidence indicates that dependence behavior leads to an excessive reinforcement of the habitual network. In our results, we did not observe the reducing connectivity of the goal‐directed network with the dlPFC. Conversely, the results revealed a reduction in the connection between the caudate nucleus and PCUN and an increased connection between the putamen and PFC and SMA. PCUN is a part of the default mode network that can direct attention and episodic memory retrieval during the execution of goal‐directed movements (Cavanna & Trimble, 2006). It also participates in the process of self‐consciousness and self‐referential that can relate information to the self (Northoff et al., 2006). Compared with the control group, the tobacco dependence group showed decreased connectivity between the goal‐directed network and PCUN. This finding indicates that the ability for attention to the outside world and self‐connection decreases during the implementation of goal‐directed network in tobacco dependence. In the smoking group, we found an increase in the FC between the SMA and putamen. The SMA– striatum loop represents a more direct stimulus–response pathway than the prefrontal–striatum loop (Balleine et al., 2009). Thus, the defectively inhibited and repeated smoking behavior in tobacco dependence may be underpinned by enhanced FC within putamen–SMA circuits. In our study, the enhanced FC in PFC–putamen loops may contribute to dependent behaviors, such that the response may not be directly triggered by the stimulus but may be mediated by abnormal activity in PFC.

The observation about the shift of the dual system in dependent behavior may be relevant particularly to cIMT. In our study, the bilateral connectivity between the caudate and inferior frontal gyrus showed a significant negative correlation with the cIMT, and no positive correlation was observed with cIMT in the brain region that connects to the caudate. However, for the putamen, increased connectivity with the inferior temporal gyrus and MFG was strongly associated with the high cIMT. This result indicates that tobacco dependence behavior is likely to show a reduction in goal‐directed network connectivity and an enhanced connection in habit network in patients with atherosclerosis. Cognitive studies have shown that a variety of cognitive domains are associated with carotid cIMT (Mergeani et al., 2015; Wendell et al., 2016). However, complex attention, learning and memory, and executive function have been considered especially susceptible domains (van der Flier et al., 2018). Our findings are consistent with this conclusion. They confirm that carotid atherosclerosis is associated with enhanced habitual brain networks in tobacco‐dependent individuals. In the study on the relationship between age and changes in brain activity, the activity of medial frontal and parietal regions increased linearly with age during task execution, whereas task‐related activation of dlPFC decreased with age (Grady et al., 2006). Numerous factors contribute to this outcome, but our results support studies and show atherosclerosis as one of such factors.

One limitation of this study is the lack of longitudinal studies that compared the dual‐system shift in tobacco dependence of young individuals compared with those in older adults, which further proves the relationship between atherosclerosis and dual‐system changes. Therefore, further research involving tobacco dependence of a young group is needed to confirm our results. Moreover, we did not further compare the smoking groups to determine the relationship between different degrees of atherosclerosis and dual‐system brain networks. We did not further analyze the causal relationship between carotid atherosclerosis and the transformation of dual‐system brain networks, which will be further explored in future studies.

## 5 CONCLUSIONS

In conclusion, rs‐fMRI was used to investigate the relationship between tobacco dependence behavior, carotid atherosclerosis, and brain function. The results indicated that the formation of tobacco dependence behavior is related to the transformation of the dual‐system. Carotid atherosclerosis was associated with a shift in FC from a goal‐directed network to a habit network in tobacco dependence. These findings provide insights into the interplay between carotid atherosclerosis and the balance of goal‐directed and habitual networks in tobacco dependence. Although our results require replication in other samples, the relationship between cIMT and the shift of the dual system must be studied to understand the cumulative effects of vascular disease on the brain. Our findings will help future investigations of the effects of the central nervous system on behavioristics and subclinical atherosclerosis.

## CONFLICT OF INTEREST STATEMENT

The author declares that there is no conflict of interest that could be perceived as prejudicing the impartiality of the research reported.

### PEER REVIEW

The peer review history for this article is available at https://publons.com/publon/10.1002/brb3.3059.

## Data Availability

The data that support the findings of this study are available from the corresponding author upon reasonable request.
